# The influence of interstitial cells of Cajal loss and aging on slow wave conduction velocity in the human stomach

**DOI:** 10.14814/phy2.14659

**Published:** 2020-12-23

**Authors:** Tim Hsu‐Han Wang, Timothy R. Angeli, Shunichi Ishida, Peng Du, Armen Gharibans, Niranchan Paskaranandavadivel, Yohsuke Imai, Taimei Miyagawa, Thomas L. Abell, Gianrico Farrugia, Leo K. Cheng, Gregory O’Grady

**Affiliations:** ^1^ Department of Surgery University of Auckland Auckland New Zealand; ^2^ Auckland Bioengineering Institute University of Auckland Auckland New Zealand; ^3^ Graduate School of Engineering Kobe University Kobe Japan; ^4^ Graduate School of Science and Technology Hirosaki University Hirosaki Japan; ^5^ Division of Gastroenterology University of Louisville Louisville KY USA; ^6^ Division of Gastroenterology & Hepatology Mayo Clinic Rochester MN USA

**Keywords:** aging, computational simulation, human, interstitial cells of Cajal (ICC), slow wave velocity, stomach

## Abstract

Loss of interstitial cells of Cajal (ICC) has been associated with gastric dysfunction and is also observed during normal aging at ~13% reduction per decade. The impact of ICC loss on gastric slow wave conduction velocity is currently undefined. This study correlated human gastric slow wave velocity with ICC loss and aging. High‐resolution gastric slow wave mapping data were screened from a database of 42 patients with severe gastric dysfunction (*n* = 20) and controls (*n* = 22). Correlations were performed between corpus slow wave conduction parameters (frequency, velocity, and amplitude) and corpus ICC counts in patients, and with age in controls. Physiological parameters were further integrated into computational models of gastric mixing. *Patients*: ICC count demonstrated a negative correlation with slow wave velocity in the corpus (i.e., higher velocities with reduced ICC; *r* ^2^ = .55; *p* = .03). ICC count did not correlate with extracellular slow wave amplitude (*p* = .12) or frequency (*p* = .84). *Aging*: Age was positively correlated with slow wave velocity in the corpus (range: 25–74 years; *r* ^2^ = .32; *p* = .02). Age did not correlate with extracellular slow wave amplitude (*p* = .40) or frequency (*p* = .34). Computational simulations demonstrated that the gastric emptying rate would increase at higher slow wave velocities. ICC loss and aging are associated with a higher slow wave velocity. The reason for these relationships is unexplained and merit further investigation. Increased slow wave velocity may modulate gastric emptying higher, although in gastroparesis other pathological factors must dominate to prevent emptying.

## Graphical abstract

Loss of interstitial cells of Cajal and aging is associated with a higher gastric slow wave velocity. This study used human data for correlation and computational models to determine the effects on gastric emptying and mixing. The reason for these relationships is unexplained and merit further investigation.

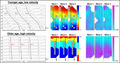

## 1 INTRODUCTION

Gastric contractions are coordinated by bioelectrical slow waves, which are generated by interstitial cells of Cajal (ICC; Huizinga & Lammers, 2009). Studies have demonstrated that loss and/or damage to ICC is a pathological hallmark of gastric dysfunction in gastroparesis and chronic unexplained nausea and vomiting (Angeli et al., 2015; Grover et al., 2011, 2012). Loss of gastric and colonic ICC has also been recognized to occur in conjunction with normal aging, at a rate of ~13% per decade, potentially contributing to the loss of functional capacity (Gomez‐Pinilla et al., 2011). However, the influence of ICC loss and aging on gastric slow wave conduction is still largely undefined.

High‐resolution (HR) gastric mapping enables detailed investigations into gastric slow wave activity (O'Grady et al., 2018). HR mapping involves the application of dense electrode arrays in order to track the detailed conduction of slow waves (Du et al., 2009; Lammers et al., 2008). This technique has allowed a better understanding of normal slow wave conduction and has characterized the pathophysiology of slow wave dysrhythmias in dysmotility states (Angeli et al., 2015; Gharibans et al., 2019; O'Grady, Angeli, et al., 2012).

A recent study by Wang et al investigated the relationship between slow wave velocity and frequency using a joint experimental‐theoretical approach (Wang et al., 2018). However, it remains unclear how these slow wave conduction parameters are impacted by ICC loss and aging. In this study, we evaluated the associations of slow wave velocity, extracellular amplitude, and frequency, derived from HR mapping techniques, with ICC count in patients, and with age in controls. Computational simulations were also performed to investigate how changes in slow wave velocity impact gastric mixing and emptying.

## 2 MATERIALS AND METHODS

Ethical approval was obtained from our Institutional Review Committees.

### 2.1 High resolution intra‐operative mapping and analysis

Human data for this study were pooled from a database of previous intra‐operative recordings performed in 20 patients with severe gastric dysfunction (including 13 patients with gastroparesis and 7 patients with chronic unexplained nausea and vomiting) and 22 controls published elsewhere (which include those undergoing elective hepatopancreaticobiliary procedures such as liver resection and pancreatic surgery; Angeli et al., 2015; Berry et al., 2016; O'Grady, Angeli, et al., 2012; Wang et al., 2018). Written consent was obtained from all subjects. All correlation analyses performed in this study were novel and unique to this study, and had not previously been performed.

Data were collected using flexible‐printed‐circuit arrays (FlexiMap; Angeli et al., 2013; Du et al., 2009) with an inter‐electrode spacing of 4 mm, which were placed intra‐operatively, over the gastric serosal surface. Patients were undergoing the implantation of gastric electrical stimulation devices; controls were undergoing non‐gastric upper gastrointestinal surgery. The arrays were connected to an ActiveTwo acquisition system modified for passive recordings (BioSemi) with a sampling rate of 512 Hz, then downsampled to 30 Hz for data analysis. The signals were filtered using a moving‐median with a window of 20 s, Savitzky–Golay filter with a low pass cut‐off frequency of 2 Hz as described and validated elsewhere (Paskaranandavadivel et al., 2013).

**FIGURE 1:**
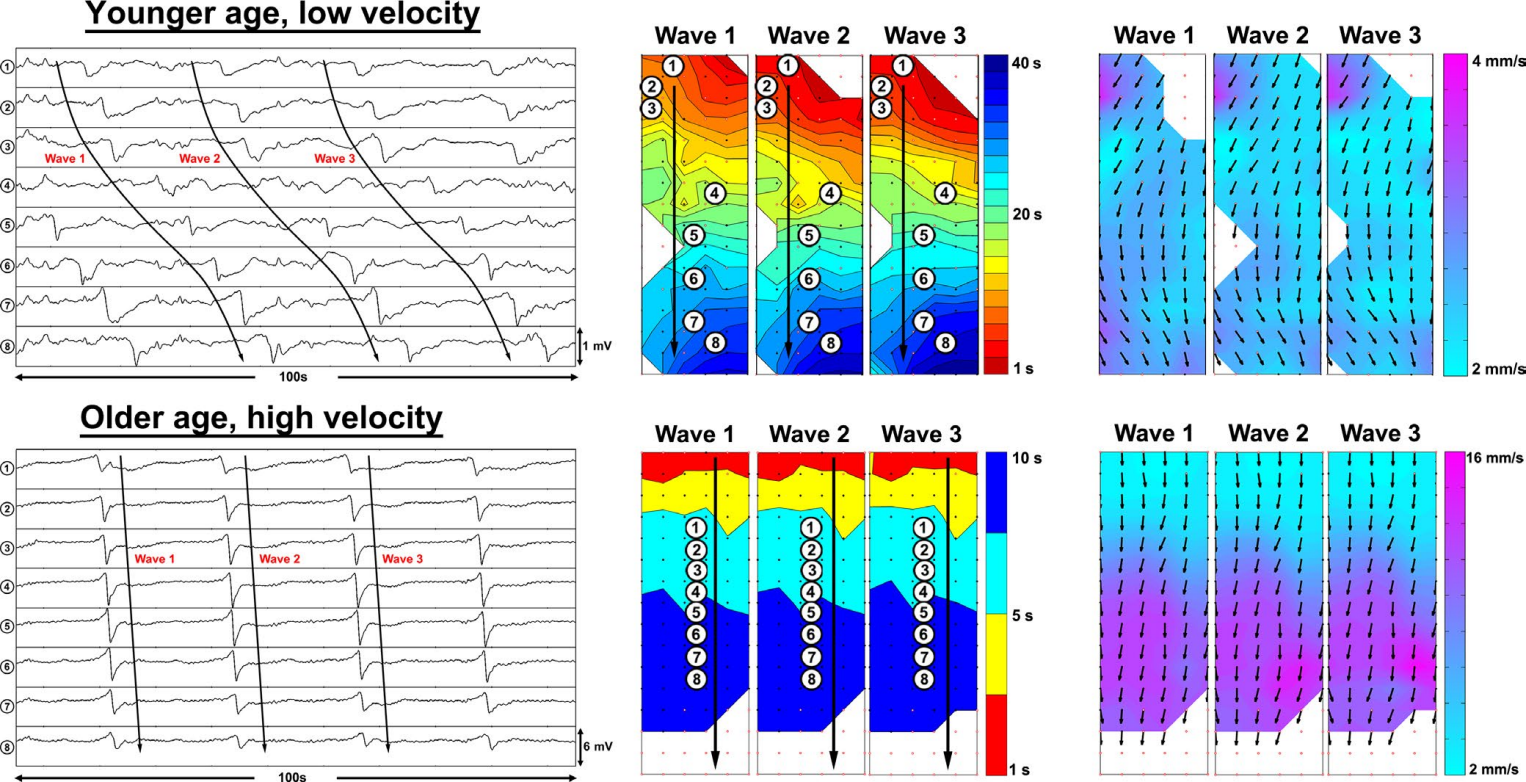
Electrograms (left) of control patients with younger (25 years old) and older age (46 years old), along with their respective activation (middle) and velocity field maps (right). Each small dot on the maps represents a single electrode. Individual electrodes were selected along the path of propagation. The inter‐electrode distance shown is 4 mm. Each color band in the activation maps represent the area of slow wave propagation per unit of time. The difference in map scales reflects the slow wave velocity differences

All HR data were analyzed in the Gastrointestinal Electrical Mapping Suite (GEMS v1.6; FlexiMap; Yassi et al., 2012). Detection of slow wave events and clustering into wavefronts was performed using semi‐automated validated algorithms with manual review and correction (Erickson et al., 2011; Erickson et al, 2010; analysis example Figure 1). Slow wave activation times, velocities, frequencies, and extracellular amplitudes were calculated and spatially mapped. Specifically, propagation velocities were calculated using a validated smooth finite‐difference method (Paskaranandavadivel et al., 2012), while extracellular slow wave amplitudes were calculated using the “zero‐crossing” of the first and second signal derivatives (Paskaranandavadivel et al., 2011).

Only gastric corpus data were analyzed, as defined by the position of the angularis incisura, because there is a transition to rapid high‐amplitude slow wave activity in the terminal human antrum that would confound results (Berry et al., 2016). It is important to note that this change in activity occurs in a relatively narrow band, approximately 28 mm from the pylorus, whereas the remainder of the corpus and antrum show a consistent velocity close to 3 mm/s (Berry et al., 2016). The direction of slow wave propagation was defined for each patient, and only antegrade longitudinal conduction data were analyzed to avoid confounding the results with the rapid propagation that accompanies circumferential conduction at pacemaker sites and during dysrhythmias (O'Grady, Angeli, et al., 2012; O'Grady, Du, et al., 2012). If longitudinal propagation direction could not be accurately determined due to excessive dysrhythmic patterns in patient data (e.g., widespread aberrant or multiple competing events), then the data for that patient were excluded.

### 2.2 ICC analysis

During the original surgery in patients with severe gastric dysfunction (following HR mapping), full‐thickness gastric biopsies were collected from the anterior stomach, midway between the curvatures, and approximately 9 cm proximal to the pylorus. This site was the same as that chosen by the Gastroparesis Clinical Research Consortium (GpCRC) in their studies of cellular defects in gastroparesis (Faussone‐Pellegrini et al., 2012; Grover et al., 2011). ICC cell bodies in the circular muscle layer were identified using a Kit antibody (mouse 1:400; Lab Vision MS‐482‐P; Thermo Fisher Scientific) and a 4,6‐diamidino‐2‐phenylindole nucleus counterstain. Using the method described by Grover et al., (2011, 2012), ICC count was calculated by quantifying the number of cell bodies per field across 20–40 high‐powered fields per specimen. The same patient cohort and ICC analysis technique were used as by O’Grady et al and Angeli et al (Angeli et al., 2015; O'Grady, Angeli, et al., 2012). Due to ethical considerations, invasive biopsies were not obtained from control patients, who were undergoing abdominal surgery for other reasons.

### 2.3 Statistical correlations

The means of slow wave velocity, extracellular amplitude, and frequency were calculated for each cycle of activity, and then the average of these individual cycles was calculated as mean ± standard error of the mean for each subject. Two correlation analyses were then completed. The first analysis, performed in the patient cohort, correlated ICC count with their slow wave characteristics, including velocity, extracellular slow wave amplitude, and frequency. The second analysis, performed in the control cohort, correlated age with slow wave velocity, extracellular amplitude, and frequency. All analyses were performed in Prism v6 (GraphPad), using linear regression. Multiple linear regression analyses were also completed to account for the confounding effect of frequency on velocity (Wang et al., 2018). *p* values less than .05 were considered statistically significant.

### 2.4 Computational modeling

**FIGURE 2:**
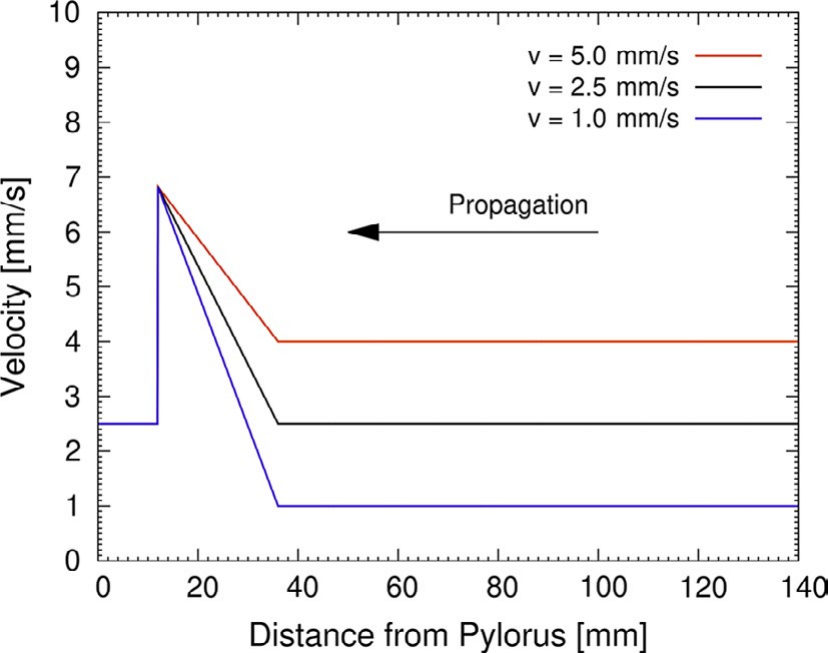
Velocity of peristaltic contraction as a function of the distance from the pylorus, as applied in computational simulations of gastric mixing and emptying

Effects of slow wave velocity on gastric mixing and emptying were quantified by a computational fluid dynamics simulation. We previously developed a three‐dimensional computational fluid dynamics model of gastric flow based on anatomically realistic gastroduodenal geometry (Berry et al., 2016; Imai et al., 2013; Ishida et al., 2019; Miyagawa et al., 2016). The cycle period of peristaltic contraction was *T* = 20 s. The pylorus began to close at the same time as the onset of terminal antral contraction and the duration of pyloric closure was *T_C_/T* = 2/3 (Berry et al., 2016). An incompressible Newtonian liquid was considered for gastric content, where the density was *ρ* = 1.0 × 10^3^ kg/m^3^ and the viscosity was *μ* = 1.25 Pa·s. The velocity of slow waves (peristaltic contractions) is given as shown in Figure 2. Gastric mixing and emptying were quantified using mixing efficiency and emptying rate as described elsewhere (Ishida et al., 2019; Miyagawa et al., 2016).

## 3 RESULTS

### 3.1 Subjects analyzed

After the exclusion of ineligible data, a total of 8 patients with severe gastric dysfunction and 17 controls were included in the analysis. From these subjects, 9,739 individual slow wave data points over 148 cycles were obtained from the patients with severe gastric dysfunction and a total of 8,318 individual slow wave data points over 210 cycles were obtained from the controls. The excluded gastric dysfunction patients (*n* = 12) were removed due to slow wave activity having dysrhythmic propagation patterns that prevented accurate velocity estimation. The excluded controls (*n* = 5) were removed due to their data being taken mostly from the distal antrum.

### 3.2 Relationship of ICC count with slow wave characteristics in patients

**FIGURE 3:**
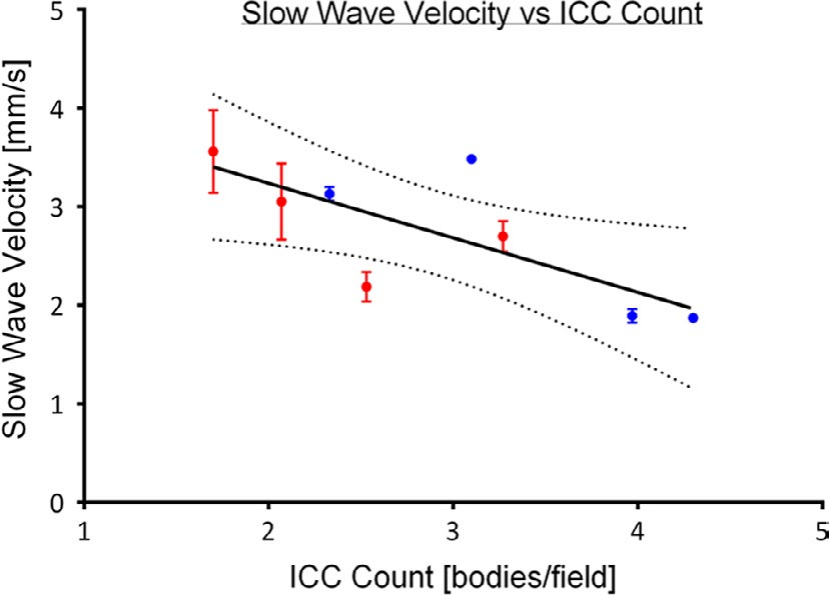
Negative linear correlation between slow wave velocity and ICC count (*r* ^2^ = .55, *p* = .03). With multiple regression *R* ^2^ = 0.60. Red plots represent patients with gastroparesis. Blue plots represent patients with chronic nausea and vomiting. Some error bars are not shown due to the margin of error being smaller than the physical size of the mean plots

ICC count demonstrated a negative correlation with longitudinal slow wave conduction velocity in the corpus (*r* ^2^ = .55), of slope 0.55 mm/s per cell body per high‐powered field (*p* = .03), meaning that pathological reduction in ICC count was correlated with faster conduction velocity (Figure 3). With multiple regression analysis to account for the effects of frequency, *R* ^2^ was 0.60. ICC count was not associated with any change in extracellular slow wave amplitude (*p* = .12) or frequency (*p* = .84). The subgroups of patients with severe gastric dysfunction were analyzed together due to the small number of total cases.

### 3.3 Relationship of age with slow wave characteristics in controls

**FIGURE 4:**
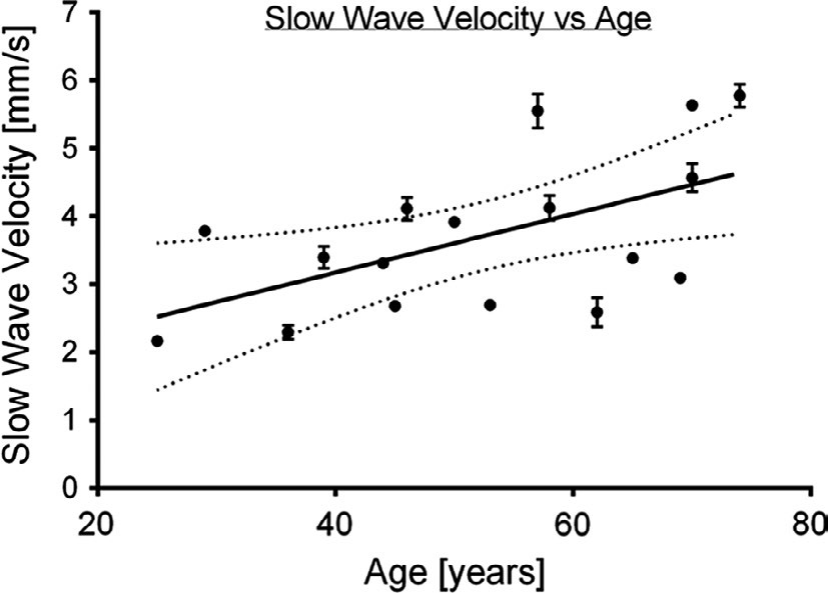
Positive linear correlation between slow wave velocity and age (*r* ^2^ = .32, *p* = .02). With multiple regression *R* ^2^ = 0.32. Some error bars are not shown due to the margin of error being smaller than the physical size of the mean plots

**FIGURE 5:**
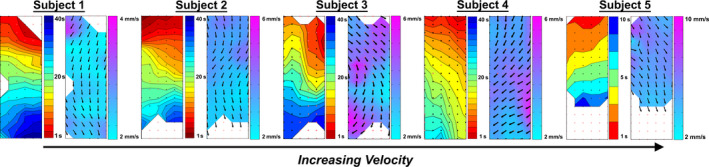
Activation and velocity field maps of five control subjects, showing a spectrum of gastric slow wave velocities observed in our cohort. The respective ages are 25, 39, 45, 58, and 74 years old (left to right). Proximal stomach is indicated at the top of all maps and the distal stomach is indicated at the bottom of all maps. The differences in both the activation and velocity map scales reflect the slow wave velocity differences

A positive correlation was identified between age and slow wave velocity in the corpus (*r* ^2^ = .32; Figure 4), of slope 0.43 mm/s per decade of age (*p* = .02). With multiple regression, *R* ^2^ was 0.32. There was no association between extracellular slow wave amplitude and age (*p* = .40), or slow wave frequency and age (*p* = .34). An example of the spectrum of slow wave velocities observed in this cohort is shown in Figure 5.

### 3.4 Computational models of gastric mixing and emptying

**FIGURE 6:**
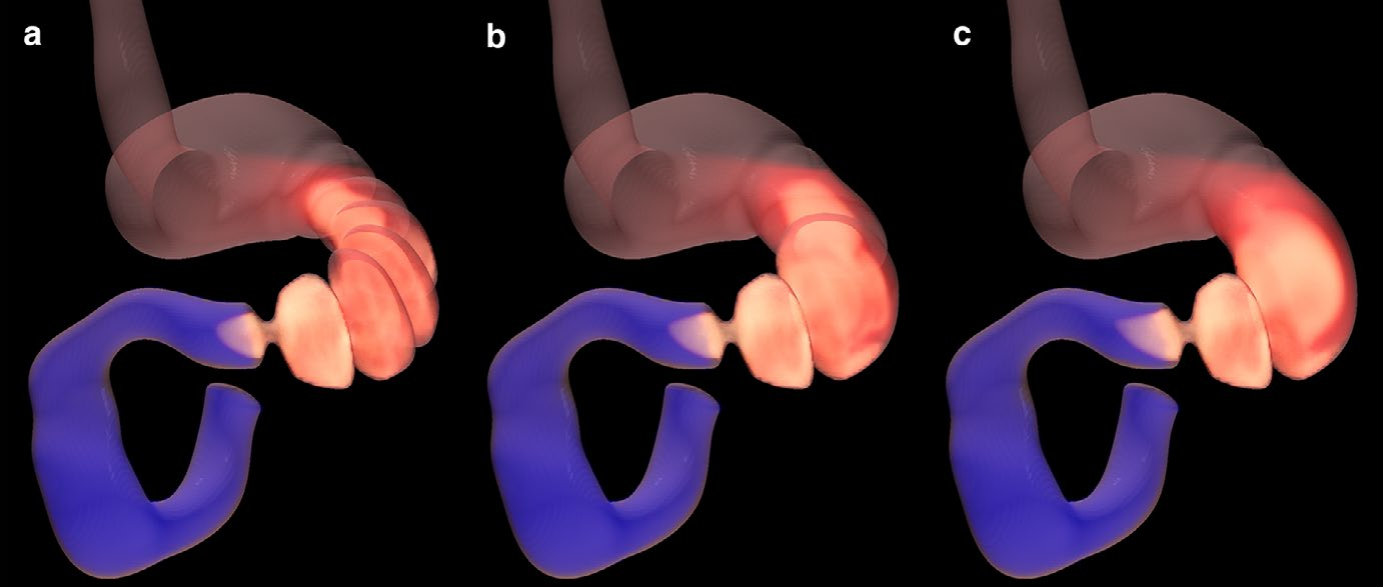
Snapshots of numerical results from the gastric contraction simulations for (a) v = 1.0 mm/s, (b) 2.5 mm/s, and (c) 5.0 mm/s

**FIGURE 7:**
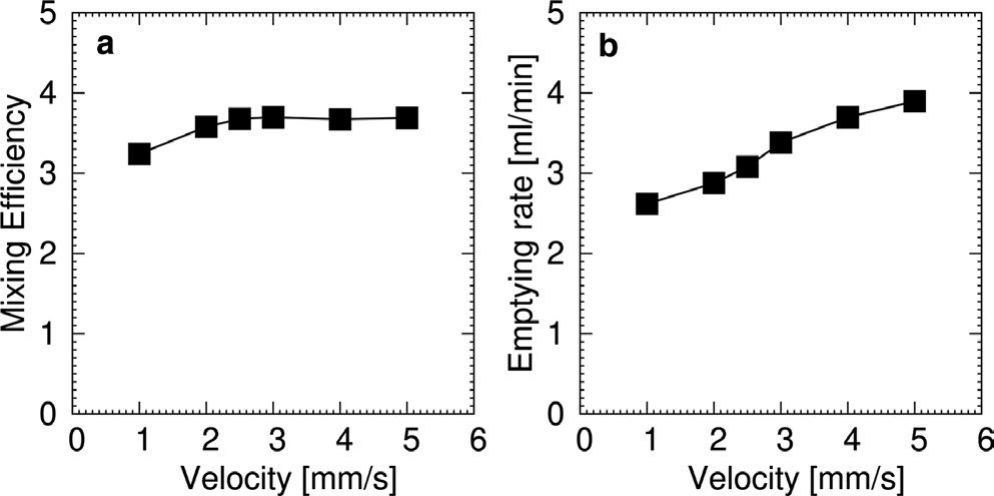
Computational simulation for (a) mixing efficiency and (b) emptying rate for a liquid content with *μ* = 1.25 Pa·s

A change in slow wave velocity alters the predicted geometry of the stomach during gastric contractions (Figure 6). When slow wave velocity is higher, the number of constrictions at an instantaneous snapshot becomes lower. Figure 6 illustrates six gastric contractions at lower slow wave velocities (1 mm/s) and two contractions at higher velocities (5 mm/s). Simulated mixing efficiency was nearly constant for the examined range of slow wave velocity (Figure 7a). However, the simulated emptying rate increased with higher slow wave velocity (Figure 7b). In the model with a low slow wave velocity of 1 mm/s, the distance of travel was 20 mm per slow wave period. At a standard slow wave velocity of 2.5 mm/s, distance of travel increased to 50 mm, and gastric mixing efficiency was 14% higher, with the gastric emptying rate being 18% higher. In the model with an elevated slow wave frequency of 5 mm/s, the distance of travel increased to 100 mm per period, and mixing efficiency remained unchanged, while gastric emptying increased another 26% over the standard model.

## 4 DISCUSSION

In this study, we defined relationships between slow wave velocity, ICC count, and aging. The key experimental findings were an inverse relationship between ICC count and slow wave velocity, and a positive correlation between age and slow wave velocity. Both of these correlations remained present or became stronger with multiple regression analysis, which accounted for the known confounding effect of slow wave frequency on conduction velocity (Wang et al., 2018).

These physiological findings were unexpected because it had previously been predicted that slow wave propagation would be slowed as ICC networks were depleted (Angeli et al., 2015; O'Grady, Angeli, et al., 2012). Biophysically based models based on isolated tissue strips from mice have also predicted that slow wave propagation would become more haphazard in depleted networks, slowing overall propagation velocity (Du et al., 2010). Animal models, albeit in the small intestines, have not demonstrated a clear association between ICC and slow wave conduction (Lammers et al., 2011). Our studies here demonstrated an opposite effect in the intact human stomach, with decreased ICC counts correlating to higher propagation velocities, suggesting that an alternative physiological mechanism is dominant in vivo. We also identified an age‐related increase in conduction velocity, which we hypothesize could similarly be related to the progressive underlying ICC loss known to occur naturally with aging (~13% per decade; Gomez‐Pinilla et al., 2011).

The specific cellular mechanisms underlying the velocity relationships defined in this study are unknown to us. The gastric ICC network represents a complex interplay between biological, electrical, and biochemical events. Compensatory plasticity within ICC networks has previously been suggested to restore motility when ICC is lost (Gao et al., 2013; Klein et al., 2013), and ICC networks have also been observed to rapidly remodel toward the dominant conduction direction during physiological pruning (Gao et al., 2014). Such compensations could impact conduction velocity, but have not yet been studied or observed in human gastric networks. It is also possible that syncytial factors beyond ICC alone could be impacting conduction velocity, such as a role for neuronal, smooth muscle, or other cell types that are coupled with and are interdependent with ICC and its network within the human stomach (Huizinga & Lammers, 2009; Huizinga et al., 2009).

One aspect of the potential functional significance of our findings was evaluated by computational simulations. These simulations found that the extent of gastric mixing was not substantially altered by slow wave velocity. As gastric mixing was mainly promoted by terminal antral contractions in conjunction with the pyloric closure, the increased slow wave velocity at the corpus had only a minimal effect. In contrast, an increase in the slow wave velocity increased the simulated gastric emptying rate. The underlying mechanism is related to the traveling distance of a peristaltic contraction; that is, a peristaltic contraction with a larger velocity travels at a longer distance during the pyloric opening, promoting greater emptying.

Associating a faster gastric emptying rate with higher contraction velocities in the context of reduced ICC may seem counter‐intuitive, because ICC loss and delayed gastric emptying are cardinal features of gastroparesis (Grover et al., 2011, 2012; O'Grady, Angeli, et al., 2012). One benefit of in silico physiological modeling is to provide an idealized theoretical environment to rigorously evaluate only specified components of a larger complex integrated system (Du et al., 2013). Therefore, other factors in gastroparesis influencing emptying such as reduced current transfer to smooth muscle cells from a reduced total ICC mass, antropyloric dysfunction, extrinsic neuropathy, gastric dysrhythmias, and injury to other cell types appear to outweigh any potential compensatory benefit arising from increasing velocity augmenting emptying (Du et al., 2010).

It is interesting to also note the increased prevalence of functional gastrointestinal symptoms with age, such as the decreased ability to eat large boluses of food, gastroesophageal reflux disease, and fecal incontinence (De Lillo & Rose, 2000). These, in part, may be contributed by the changes in ICC counts and networking, leading to changes in slow wave changes and hence changes in gastric function.

The main strength of this study was that it utilized optimal intra‐operative HR mapping techniques on human subjects to accurately identify the slow wave characteristics (O'Grady et al., 2018). Although the numbers of patients available for correlation were relatively small, once cases of antral or disorganized propagation were excluded, the number of individual data points for each patient was high due to the multi‐electrode approach (comprising >18,000 individual slow wave events). In addition, further subgroup analysis into specific disease groups and age‐decade subgroup analysis was not able to be performed in this study due to the relatively small number of total patients included. Another limitation was that biopsies were unable to be obtained from the control cohort for ethical and safety reasons, and as such, comparison between ICC count was unable to be performed between the patient and control cohorts. In the future, additional controlled studies and further subgroup analyses could be beneficial in further elucidating the significance of the physiological findings of this study, either using the HR mapping techniques or emerging non‐invasive body‐surface mapping approaches (Gharibans et al., 2017; O'Grady et al., 2018).

In conclusion, this study has identified significant relationships between human gastric slow wave conduction velocity and ICC depletion and aging. This unexpected finding awaits further evaluation and explanation.

## CONFLICT OF INTEREST

TRA, PD, AG, NP, LKC, GOG hold intellectual property in the field of gastric electrophysiology evaluation of FlexiMap Ltd. TRA, PD, NP, LKC, GOG are shareholders of FlexiMap Ltd.

## AUTHOR CONTRIBUTIONS

Concept and funding: TH‐HW, TRA, PD, GOG; Devices and experiments: TRA, SI, PD, NP, YI, TM, TLA, GF, LKC, GOG. Analysis and interpretation of results: TH‐HW and all authors; Manuscript draft and figures: TH‐HW, TRA, SI, YI, GOG. Review and approval of the manuscript: all authors.

## Data Availability

Data available on request due to privacy/ethical restrictions.
